# Association between the endothelial activation and stress index and cardiometabolic multimorbidity: Results from the NHANES 2003 to 2020

**DOI:** 10.1097/MD.0000000000048226

**Published:** 2026-04-10

**Authors:** Qiang Shi, Xiang Chen, Yifei Chen

**Affiliations:** a Yangzhou University, Yangzhou, Jiangsu, China; b Department of Emergency, Suzhou Ninth People’s Hospital, Suzhou, Jiangsu, China; c Jiangsu Key Laboratory of Zoonosis, Jiangsu Co-Innovation Center for Prevention and Control of Important Animal Infectious Diseases and Zoonoses, Yangzhou, Jiangsu, China; d Department of Emergency Medicine, Affiliated Hospital of Yangzhou University, Yangzhou University, Yangzhou, Jiangsu, China.

**Keywords:** cardiometabolic multimorbidity, cross-sectional study, Endothelial Activation and Stress Index, NHANES, U.S. adults

## Abstract

Cardiometabolic multimorbidity (CMM) poses a significant global health burden, with endothelial dysfunction being a shared pathophysiological mechanism. The Endothelial Activation and Stress Index (EASIX) is a novel and powerful marker reflecting endothelial function, but its association with CMM remains unclear. In the fully adjusted model, each unit increase in natural logarithmic transformed (Ln)-EASIX was associated with a 39% increased risk of CMM (odds ratios = 1.39, 95% confidence intervals: 1.19–1.64, *P* < .001). A nonlinear L-shaped dose–response relationship was observed in restricted cubic spline model. Threshold effect revealed a significant positive association between Ln-EASIX and CMM risk when Ln-EASIX > −0.916 (odds ratios = 1.857, 95% confidence intervals: 1.615–2.133, *P* < .001). Subgroup analyses demonstrated that hyperuricemia significantly modified the association between Ln-EASIX and CMM. The area under the curve was 0.66, showing that EASIX has good prediction ability for CMM. EASIX is significantly and positively associated with the prevalence of CMM, suggesting its potential role as a biomarker for identifying high-risk individuals and underscoring the importance of endothelial dysfunction in CMM. This study utilized data from the National Health and Nutrition Examination Survey 2003 to 2020, including 20,040 adult participants. EASIX was calculated as lactate dehydrogenase (U/L) × creatinine (mg/dL)/platelet count (10^9^/L). CMM was defined as the co-occurrence of 2 or more cardiometabolic diseases, including type 2 diabetes mellitus, myocardial infarction, and stroke. Weighted logistic regression models, restricted cubic spline regression model, subgroup analyses were employed to assess the association. The receiver operating characteristic curve and area under the curve were used to assess the predictive power of EASIX.

## 1. Introduction

Cardiometabolic multimorbidity (CMM) is defined as the co-occurrence of 2 or more cardiometabolic diseases (CMDs), including type 2 diabetes mellitus, myocardial infarction (MI), and stroke in the same individual.^[1]^ Epidemiological data reveal considerable variation in the global prevalence of CMM, with reported rates of 5.94% in China compared to 14.4% in the United States.^[2]^ Once CMM is established, life expectancy is shortened by more than 10 years compared with individuals free of any CMD.^[3]^ A graded, positive association was observed between the number of CMD and the length of hospital stay. Specifically, patients with a greater multiplicity of CMD experienced progressively longer hospitalizations compared to those with fewer or no such diagnoses, indicating a higher overall disease burden.^[4]^ Effective prevention and integrated management of CMM therefore represent a priority for contemporary health systems, but early identification of individuals at highest risk remains challenging.

CMDs share common pathophysiological pathways, with systemic endothelial dysfunction being a central, propagating mechanism that accelerates disease progression and amplifies mortality risk.^[5,6]^ The Endothelial Activation and Stress Index (EASIX), a novel and readily calculable biomarker reflecting endothelial function.^[7]^ Initially validated for predicting endothelial complications and survival post-allogeneic stem cell transplantation, the clinical relevance of this index has since transcended hematological settings, finding significant applications in diverse fields such as oncology and critical care.^[8]^ Population based epidemiological studies have found that elevated EASXI was significantly associated with the occurrence of cardiovascular events.^[9]^ Another study demonstrated the utility of the EASIX in predicting all-cause mortality among patients with asthma.^[10]^ However, the association of EASIX with CMM risk has not been revealed.

To address this gap, we investigated the association between EASIX and CMM using data from the National Health and Nutrition Examination Survey (NHANES) and conducted a large cross-sectional study. This study aims to elucidate the role of endothelial dysfunction in CMM, thereby providing scientific evidence to inform future prevention and intervention strategies.

## 2. Methods

### 2.1. The study design and population

The NHANES is a nationally representative, cross-sectional surveillance program employing a complex, multistage sampling design to monitor the health and nutritional status of the non-institutionalized U.S. population. All survey protocols were approved by an institutional review board, and written informed consent was provided by all participants.^[11]^

**Figure 1.**
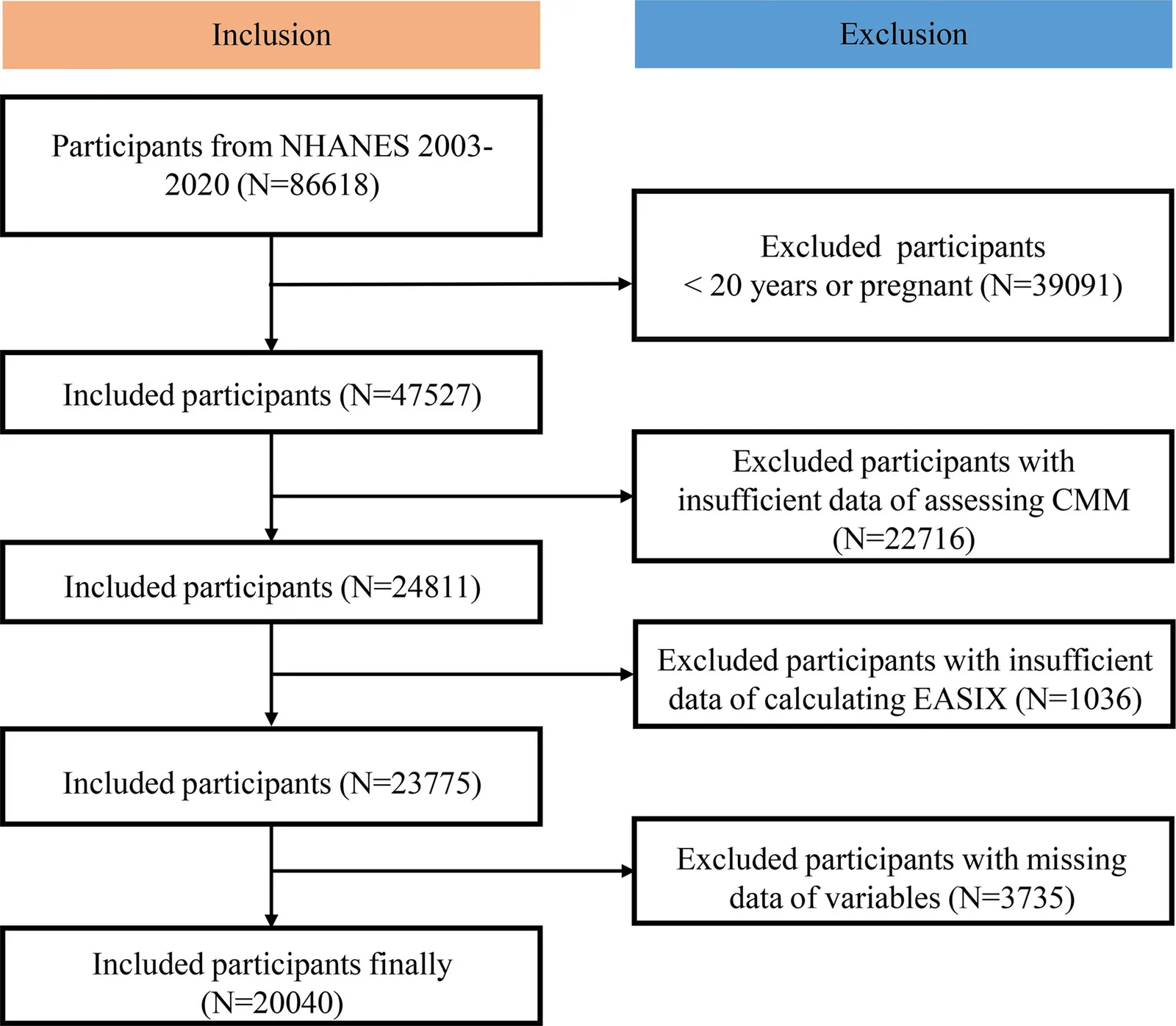
Screening flow of participants.

Based on the NHANES data from 2003 to 2020, which initially included 86,618 participants, a rigorous selection process was applied to define the final study cohort. The study first excluded 39,091 participants who were under 20 years of age or pregnant. From the remaining 47,527 individuals, a further 22,716 were excluded due to insufficient data for assessing CMM. Subsequently, 1036 participants were excluded because of insufficient data required to calculate the EASIX. Finally, an additional 3735 participants were removed due to missing data on other key variables of interest. After applying these sequential exclusion criteria, a total of 20,040 participants were retained as the final study population for analysis (Fig. 1).

### 2.2. Calculating of EASIX

The EASIX was calculated using the formula: lactate dehydrogenase (LDH, U/L) × creatinine (mg/dL)/platelet count (10^9^/L).^[9]^ All laboratory testing was strictly performed in accordance with the standardized NHANES protocol.

### 2.3. Assessment of CMM

CMM was characterized by the coexistence of CMDs, including diabetes, MI, and stroke.^[1]^ Diabetes was defined as meeting any of the following criteria: self-reported physician diagnosis; current use of glucose-lowering medications; or fasting plasma glucose ≥ 126 mg/dL or hemoglobin A1c ≥ 6.5%.^[12]^ The information for MI and stroke came from self-reported medical history. CMM was characterized by the coexistence of 2 or more CMDs.

### 2.4. Covariates

We adjusted for a comprehensive set of demographic, lifestyle, and clinical factors that are putative confounders of the EASIX–CMM association. Covariates included and adjusted for in our analyses encompass 2 broad categories: sociodemographic variables and life behavior/disease variables. Sociodemographic variables consist of sex, age, race/ethnicity, educational level, poverty-to-income ratio (PIR), and marital status. Life behavior and disease variables include body mass index (BMI), smoking status, drinking status, hypertension, hyperlipidemia, and hyperuricemia. See Table S1, Supplemental Digital Content, https://links.lww.com/MD/R619 for detailed covariate definitions.^[13–15]^

### 2.5. Statistical analysis

All statistical analyses were performed utilizing R software (version 4.5.0; R Foundation for Statistical Computing, Vienna, Austria), and a 2-tailed *P* value < .05 was considered statistically significant. Examination weights from the Mobile Examination Center were applied in our analyses. In descriptive analyses, EASIX was summarized as weighted median along with the 25th and 75th percentiles since it follows a skewed distribution. Categorical variables were exhibited as counts (weighted percentages). We contrasted the distribution differences of the variables between the CMM group and non-CMM group, employing weighted Kruskal–Wallis test for EASIX and weighted chi-square test for categorical variables. We performed natural logarithmic (Ln) transformation of EASIX to eliminate outliers and included it in subsequent analyses.^[16]^

To examine the association between EASIX and CMM, we conducted weighted logistic regression analyses to estimate odds ratios (ORs) and their corresponding 95% confidence intervals (CIs). Three sequential models were fitted: a crude model that was adjusted for no covariate (Model 1), a model adjusted for demographic factors including sex, age, race/ethnicity, educational level and PIR, and marital status (Model 2), and a fully adjusted model incorporating all covariates including sex, age, race/ethnicity, educational level and PIR, and marital status, BMI, smoking status, drinking status, hypertension, hyperlipidemia, and hyperuricemia (Model 3). EASIX was analyzed both as a continuous variable and as a categorical variable (grouped by quartiles). We also calculated McFadden Pseudo *R*^2^ for each logical regression. To assess potential nonlinear association, restricted cubic splines (RCS) regression model was employed.^[17]^ We also applied threshold effect analysis to test the significance of nonlinear association.^[18]^ Then, subgroup analyses were performed to investigate potential interaction effects between EASIX and other covariates on the prevalence of CMM.^[19]^ Finally, the diagnostic performance of EASIX in predicting CMM was assessed by the receiver operating characteristic (ROC) curve, with a larger area under the curve corresponding to a more robust predictive ability.^[20]^

## 3. Results

### 3.1. Baseline characteristics of study participants

Among the 20,040 participants, 1124 participants were identified as having CMM. Comparative analysis of baseline characteristics revealed statistically significant differences between the CMM and non-CMM groups across all variables (all *P* < .05). Specifically, participants in the CMM group were older and more likely to be males. Racial composition also differed, with the CMM group having higher proportions of non-Hispanic White and non-Hispanic Black individuals. Furthermore, the CMM group was characterized by a lower educational attainment, poorer, and a higher rate of being widowed, divorced, or separated. CMM had a higher BMI and a greater proportion of nondrinkers and current smokers. Concurrently, the prevalence of comorbidities including hypertension, hyperlipidemia, and hyperuricemia was markedly higher in the CMM group. Most critically, the median EASIX was significantly elevated in the CMM group compared to the non-CMM group (0.41 in non-CMM group and 0.59 in CMM group) (Table 1).

**Table 1:** Characteristics of the participants grouped by CMM in NHANES 2003–2020.

Variables	Overall	Non-CMM group	CMM group	*P*
n	20,040	18,916	1124	
Age, n (%)				<.001
20–39 yr	5703 (31.9)	5684 (33.2)	19 (2.2)	
40–59 yr	6732 (39.0)	6512 (39.5)	220 (25.3)	
≥60 yr	7605 (29.1)	6720 (27.2)	885 (72.5)	
Gender, n (%)				.001
Male	10,026 (49.7)	9346 (49.4)	680 (56.9)	
Female	10,014 (50.3)	9570 (50.6)	444 (43.1)	
Race, n (%)				<.001
Mexican American	3153 (8.0)	3020 (8.2)	133 (4.4)	
Other Hispanic	1765 (5.1)	1685 (5.2)	80 (3.3)	
Non-Hispanic White	8792 (69.0)	8233 (68.8)	559 (73.0)	
Non-Hispanic Black	4245 (10.5)	3965 (10.4)	280 (12.9)	
Other Race – including multi-racial	2085 (7.4)	2013 (7.4)	72 (6.4)	
Education, n (%)				<.001
Less than high school	5067 (16.2)	4653 (15.8)	414 (27.1)	
High school graduate/GED or equivalent	4634 (23.7)	4345 (23.4)	289 (31.7)	
Higher than high school	10,339 (60.1)	9918 (60.9)	421 (41.2)	
PIR, n (%)				<.001
≤1.3	6267 (20.7)	5806 (20.3)	461 (29.4)	
1.3–3.5	7736 (36.6)	7281 (36.2)	455 (43.9)	
>3.5	6037 (42.7)	5829 (43.4)	208 (26.8)	
Marital status, n (%)				<.001
Married/living with partner	12,066 (64.5)	11,444 (64.6)	622 (61.7)	
Widowed/divorced/separated	4726 (19.5)	4296 (18.8)	430 (33.4)	
Never married	3248 (16.1)	3176 (16.6)	72 (4.9)	
BMI, n (%)				<.001
<25 kg/m^2^	5329 (27.8)	5179 (28.4)	150 (12.8)	
25–30 kg/m^2^	6543 (32.5)	6225 (32.9)	318 (24.5)	
≥30 kg/m^2^	8168 (39.7)	7512 (38.7)	656 (62.6)	
Smoking status, n (%)				<.001
Non smokers	10,698 (53.2)	10,278 (54.0)	420 (35.9)	
Former smokers	5324 (27.0)	4837 (26.3)	487 (42.9)	
Current smokers	4018 (19.8)	3801 (19.7)	217 (21.2)	
Drinking status, n (%)				<.001
Nondrinkers	7585 (31.0)	6926 (30.0)	659 (53.9)	
Current moderate drinkers	11,230 (61.6)	10,795 (62.5)	435 (41.9)	
Current heavy drinkers	1225 (7.4)	1195 (7.5)	30 (4.2)	
Hypertension, n (%)				<.001
Yes	8969 (38.8)	8037 (37.0)	932 (81.0)	
No	11,071 (61.2)	10,879 (63.0)	192 (19.0)	
Hyperlipidemia, n (%)				<.001
Yes	15,249 (74.5)	14,177 (73.5)	1072 (96.3)	
No	4791 (25.5)	4739 (26.5)	52 (3.7)	
Hyperuricemia, n (%)				<.001
Yes	4471 (21.3)	4063 (20.7)	408 (34.0)	
No	15,569 (78.7)	14,853 (79.3)	716 (66.0)	
EASIX, median (25th, 75th)	0.41 (0.29–0.56)	0.41 (0.29–0.56)	0.54 (0.34–0.81)	<.001

BMI = body mass index, CMM = cardiometabolic multimorbidity, EASIX = endothelial activation and stress index, n = number, PIR = poverty-to-income ratio.

### 3.2. Association of EASIX with CMM risk

In Model 1, Ln-EASIX as a continuous variable was significantly associated with CMM risk, with an OR (95% CI) of 2.58 (95% CI: 2.18–3.05, *P* < .001). When analyzed by quartiles, participants in the Q4 had a 192% significantly increased risk of CMM compared to those in the Q1 (OR = 2.92, 95% CI: 2.34–3.65, *P* < .001), and a significant dose–response relationship was observed *(P* for trend < .001). After adjusting for demographic factors in Model 2, Ln-EASIX was also significantly associated with higher CMM risk (OR = 1.48, 95% CI: 1.24–1.75, *P* < .001). Quartile analysis indicated a 28% increased risk in Q4 versus Q1 (OR = 1.28, 95% CI: 1.03–1.60, *P* = .028), with a persistent significant dose–response trend (*P* for trend = .008). In Model 3, Ln-EASIX still remained an obvious positive relationship with CMM (OR = 1.39, 95% CI: 1.19–1.64, *P* < .001). Quartile analysis indicated a 23% decreased risk in Q3 versus Q1 (OR = 0.77, 95% CI: 0.60–1.00, *P* = .046). The overall test for trend remained statistically significant (*P* for trend = .018), suggesting a positive association between Ln-EASIX and CMM risk (Table 2).

**Table 2:** Weighted logistic regression models about the associations between EASIX and CMM.

	Model 1		Model 2		Model 3	
	OR (95% CI)	*P*	OR (95% CI)	*P*	OR (95% CI)	*P*
Continuous	2.58 (2.18–3.05)	<.001	1.48 (1.24–1.75)	<.001	1.39 (1.19–1.64)	<.001
Q1	Reference				Reference	
Q2	0.96 (0.73–1.25)	.738	0.81 (0.62–1.05)	.115	0.79 (0.60–1.05)	.099
Q3	1.2 (0.93–1.54)	.163	0.77 (0.60–1.00)	.052	0.77 (0.60–1.00)	.046
Q4	2.92 (2.34–3.65)	<.001	1.28 (1.03–1.60)	.028	1.21 (0.99–1.49)	.061
*P* for trend	<.001		.008		.018	

Model 1 was unadjusted.

Model 2 was adjusted for age, sex, race/ethnicity, education, PIR, and marital status.

Model 3 was adjusted for age, sex, race/ethnicity, education, PIR, marital status, BMI, smoking status, drinking status, hypertension, hyperlipidemia, and hyperuricemia.

BMI = body mass index, CI = confidence interval, CMM = cardiometabolic multimorbidity, EASIX = endothelial activation and stress index, OR = odds ratio, PIR = poverty-to-income ratio.

**Figure 2.**
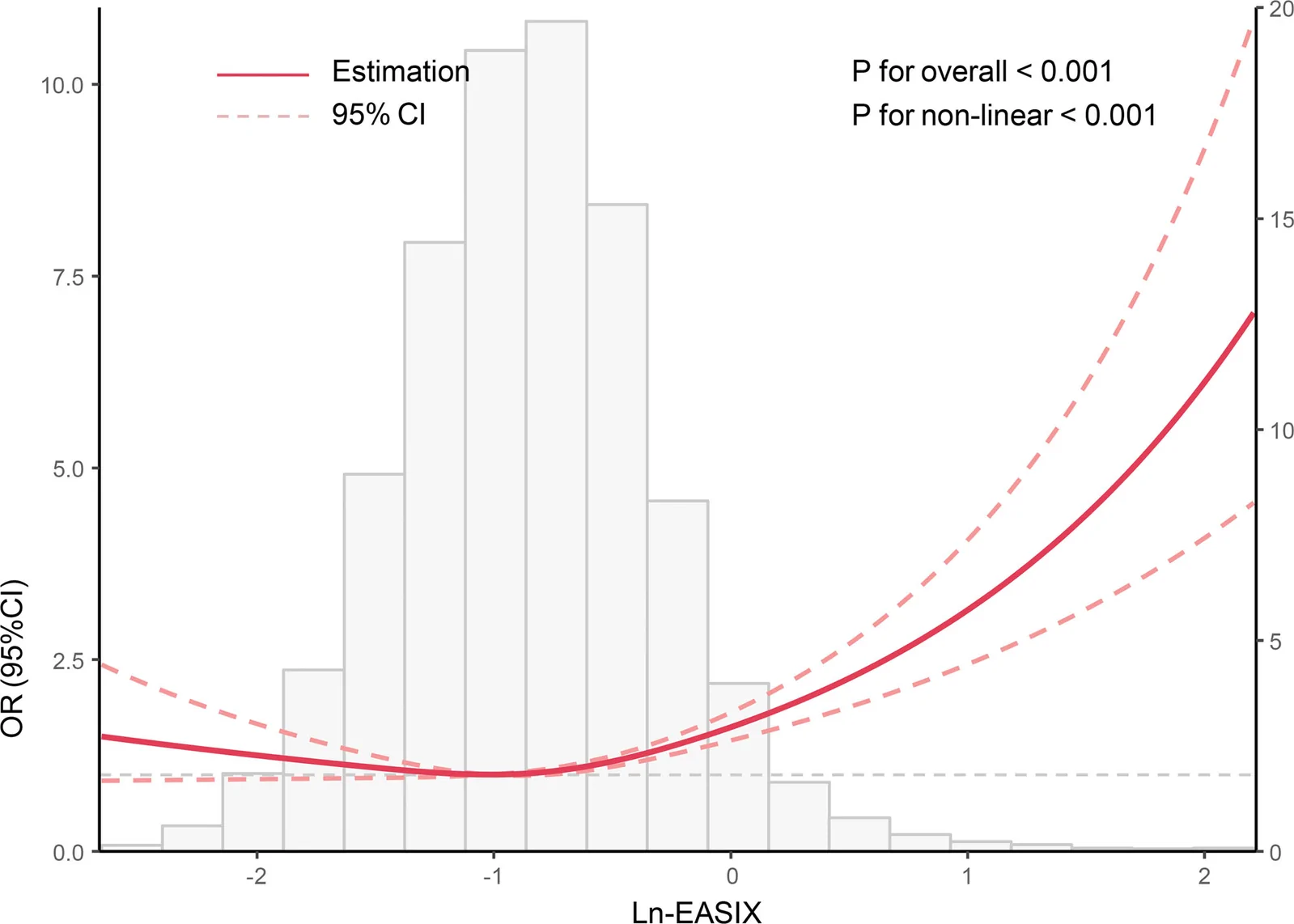
The association between Ln-EASIX and CMM in RCS regression model. Model was adjusted for age, sex, race/ethnicity, education, PIR, marital status, BMI, smoking status, drinking status, hypertension, hyperlipidemia and hyperuricemia. Bars represent the distribution of independent variables; red lines represent dose–response relationships; dashed lines represent CI; the horizontal reference line represents OR = 1. BMI = body mass index; CI = confidence interval; CMM = cardiometabolic multimorbidity; EASIX = endothelial activation and stress index; OR = odds ratio; PIR = poverty-to-income ratio; RCS = restricted cubic splines.

The results of RCS regression model were displayed in Figure 2. After adjusting for all covariates, Ln-EASIX had a nonlinear L-shaped association with the risk of CMM (*P* for overall < .001 and *P* for nonlinear < .001).

Furthermore, a threshold effect analysis identified a critical inflection point for Ln-EASIX at −0.916. When Ln-EASIX exceeded this threshold, it demonstrated an insignificant positive association with CMM risk (OR = 1.857, 95% CI: 1.615–2.133, *P* < .001), whereas no significant association was observed below this threshold (Table 3).

**Table 3:** Threshold effect analysis of Ln- EASIX and CMM correlation.

Ln-EASIX	OR (95% CI)	*P*
≤−0.916	0.749 (0.553–1.027)	.067
>−0.916	1.857 (1.615–2.133)	<.001
Log likelihood ratio test		<.001

Model was adjusted for age, sex, race/ethnicity, education, PIR, marital status, BMI, smoking status, drinking status, hypertension, hyperlipidemia and hyperuricemia.

BMI = body mass index, CI = confidence interval, CMM = cardiometabolic multimorbidity, EASIX = endothelial activation and stress index, OR = odds ratio, PIR = poverty-to-income ratio.

### 3.3. Subgroup analyses

Subgroup analyses further confirmed the consistency of this association across populations defined by age, gender, race, and other characteristics. The positive associations between Ln-EASIX and CMM remained significant in nearly all subgroups. There were no significant interactions observed, except for the hyperuricemia subgroup (*P* for interaction = .045). The association was notably stronger among individuals with hyperuricemia (OR = 1.82, 95% CI: 1.42–2.33, *P* < .001), while the association was not obvious in the individuals without hyperuricemia (Table 4).

**Table 4:** Subgroup analyses of the associations between Ln-EASIX and CMM.

Characteristic	OR (95%CI)	*P*	*P* for interaction
Age			.386
20–39 yr	0.67 (0.16–2.70)	.570	
40–59 yr	1.49 (0.96–2.32)	.078	
≥60 yr	1.39 (1.18–1.64)	<.001	
Gender			.363
Male	1.44 (1.16–1.79)	.001	
Female	1.35 (1.02–1.78)	.036	
Race			.804
Mexican American	1.43 (0.99–2.06)	.06	
Other Hispanic	1.44 (0.91–2.27)	.123	
Non-Hispanic White	1.35 (1.10–1.66)	.005	
Non-Hispanic Black	1.57 (1.27–1.94)	<.001	
Other Race – including multi-racial	1.46 (0.86–2.47)	.159	
Education			.984
Less than high school	1.43 (1.08–1.89)	.013	
High school graduate/GED or equivalent	1.38 (0.91–2.07)	.131	
Higher than high school	1.38 (1.12–1.71)	.003	
PIR			.931
≤1.3	1.46 (1.14–1.88)	.004	
1.3–3.5	1.42 (1.12–1.81)	.005	
>3.5	1.28 (0.87–1.89)	.212	
Marital status			.548
Married/living with partner	1.31 (1.05–1.64)	.019	
Widowed/divorced/separated	1.51 (1.18–1.92)	.001	
Never married	1.65 (1.00–2.72)	.05	
BMI			.764
<25 kg/m^2^	1.14 (0.63–2.06)	.665	
25–30 kg/m^2^	1.29 (1.01–1.65)	.040	
≥30 kg/m^2^	1.49 (1.21–1.85)	<.001	
Smoking status			.175
Non smokers	1.39 (1.13–1.71)	.002	
Former smokers	1.53 (1.17–2.01)	.003	
Current smokers	1.09 (0.67–1.76)	.741	
Drinking status			.745
nondrinkers	1.46 (1.18–1.79)	.001	
Current moderate drinkers	1.36 (1.04–1.78)	.028	
Current heavy drinkers	0.77 (0.22–2.68)	.687	
Hypertension			.424
Yes	1.37 (1.15–1.64)	<.001	
No	1.54 (1.00–2.36)	.052	
Hyperlipidemia			.389
Yes	1.37 (1.17–1.60)	<.001	
No	2.06 (1.19–3.57)	.011	
Hyperuricemia			.045
Yes	1.82 (1.42–2.33)	<.001	
No	1.19 (0.91–1.54)	.204	

Models were adjusted for age, sex, race/ethnicity, education, PIR, marital status, BMI, smoking status, drinking status, hypertension, hyperlipidemia and hyperuricemia.

BMI = body mass index, CI = confidence interval, CMM = cardiometabolic multimorbidity, EASIX = endothelial activation and stress index, OR = odds ratio, PIR = = poverty-to-income ratio.

### 3.4. ROC curve of the predicted performance of EASIX

**Figure 3.**
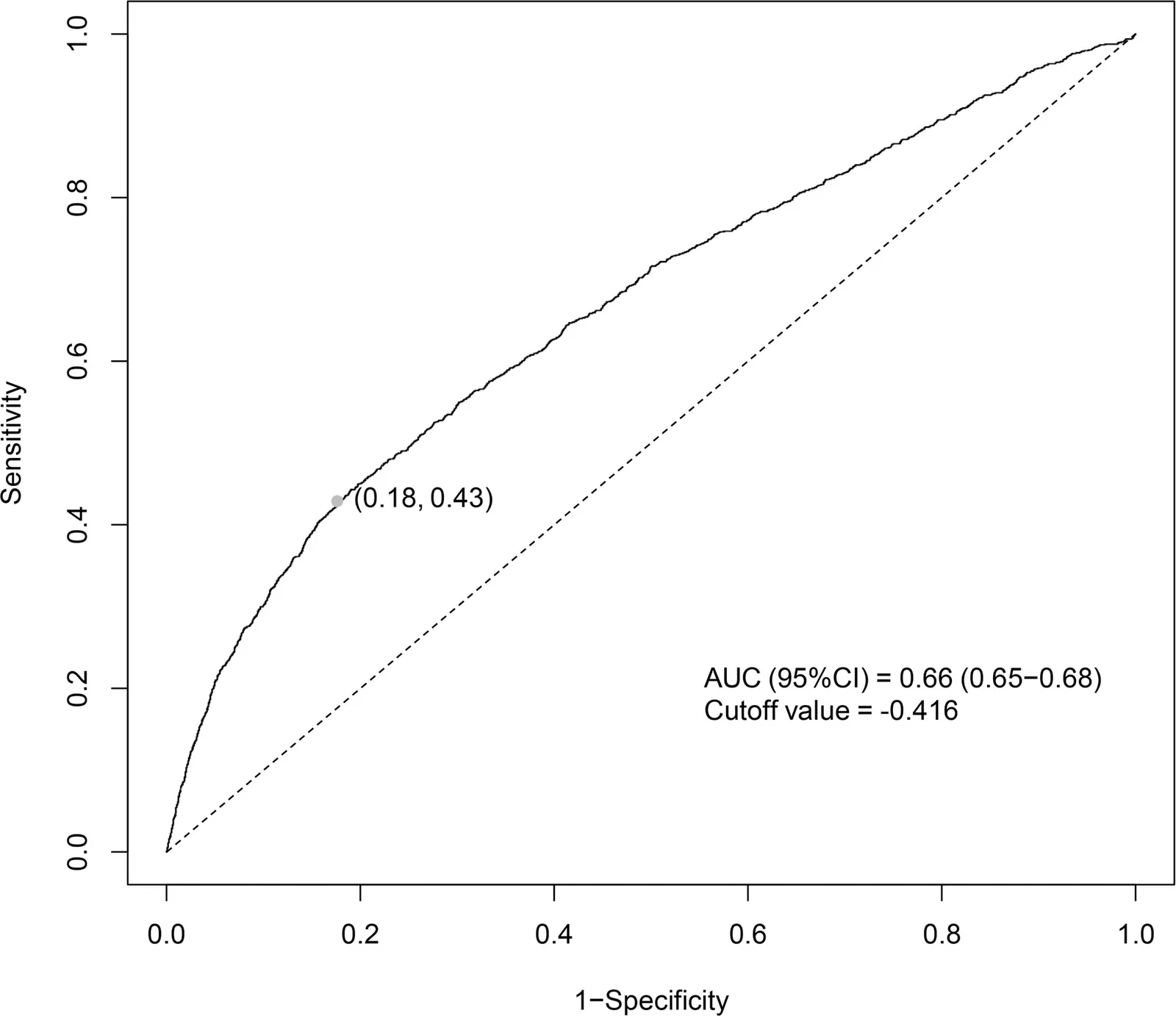
ROC curve for the predictive power of Ln-EASIX. AUC = area under the curve; CI = confidence interval; CMM = cardiometabolic multimorbidity; EASIX = endothelial activation and stress index; OR = odds ratio; ROC = receiver operating characteristic.

The discriminative ability of Ln-EASIX for CMM was assessed using ROC curve analysis (Fig. 3). The model achieved an AUC of 0.66 (95% CI: 0.65–0.68), indicating a statistically significant but modest predictive accuracy. At the optimal cutoff point of −0.416, the model yielded a sensitivity of 43% and a specificity of 82%. These results showed that EASIX has good predictive ability for CMM.

## 4. Discussion

Utilizing a large, nationally representative sample, this study is the first to investigate the association between the EASIX and CMM. Our primary finding is that a higher EASIX level was independently and significantly associated with an increased prevalence of CMM. Furthermore, we observed a nonlinear, L-shaped dose–response relationship between EASIX and CMM risk, identifying a specific threshold effect. Subgroup analyses further confirmed the consistency of this association across most predefined subgroups, with a notably stronger effect observed among individuals with hyperuricemia. ROC curves indicated that EASIX had a better predictive performance of CMM. Collectively, these findings suggest that EASIX, serving as a surrogate marker of endothelial dysfunction and systemic stress, may be a promising biomarker for identifying individuals at high risk for CMM.

The findings of our study are grounded in solid pathophysiological rationale. The core shared pathways of CMD include chronic inflammation, oxidative stress, and most critically, endothelial dysfunction.^[21–23]^ The EASIX formula integrates LDH, creatinine, and platelet count, which collectively reflect different facets of the endothelial injury process: LDH is a marker of cellular damage and necrosis, potentially released upon vascular endothelial ischemia or inflammatory injury; renal impairment (elevated creatinine) accelerates endothelial dysfunction; and alterations in platelet count may relate to consumption or activation following endothelial damage.^[24–26]^ It is worth noting that threshold effect analysis showed that low level EASIX is not significantly associated with CMM risk. When EASIX is at low water level, the slight endothelial activation or cell stress it represents is completely within the scope of the body’s own regulation and repair ability.^[27]^ Under the stress represented by low levels of EASIX, intracellular defense mechanisms (such as antioxidants) may be effectively up-regulated, thus maintaining the homeostasis.^[28]^ Therefore, as a composite index, EASIX likely captures the disordered vascular endothelial microenvironment that promotes the development and progression of CMM more comprehensively than any single biomarker.

Our results align with emerging epidemiological evidence. For instance, Liu et al found that EASIX was associated with an increased risk of CVD in the general U.S. population; our study extends this association to the more complex phenotype of CMM.^[9]^ Their conclusions, however, reveal only an association between EASIX and a single CVD. An important incremental contribution of this study is that it reveals the association between EASIX and the coexistence of multiple diseases, suggesting that monitoring the state of endothelial inflammation is not only helpful in predicting a single event, but may be of unique value in identifying individuals who are about to enter a high-risk stage of multiple disease burdens. Another study on NHANES showed that EASIX was a reliable predictor of stroke prevalence and mortality in stroke population.^[29]^ Our results suggest that EASIX is equally effective in predicting this more complex clinical trajectory than the endpoint of mortality after stroke, which represents a chronic, progressive multiple disease burden. Furthermore, EASIX demonstrated significant positive associations with diabetic retinopathy, all-cause mortality, and CVD mortality.^[30,31]^ It is noteworthy that the association between EASIX and CMM was particularly pronounced in individuals with hyperuricemia. This effect modification has a plausible biological explanation, as hyperuricemia itself can induce oxidative stress, inflammatory responses, and direct endothelial injury, potentially acting synergistically with the endothelial stress represented by EASIX to markedly amplify the risk of CMM.^[32,33]^ More research is needed to confirm our conclusion in the future.

This study has several notable strengths. First, to our knowledge, it represents the first large-scale epidemiological investigation to explore the association between the EASIX and CMM within a nationally representative sample. Second, the utilization of high-quality data from the NHANES, with its complex sampling design and stringent quality control protocols, ensures data reliability and enhances the generalizability of our findings to the non-institutionalized U.S. population. Finally, we employed rigorous statistical methodologies, including weighted logistic regression models, complemented by RCS regression model and threshold effect analysis to delineate the shape of the exposure–response relationship, thereby strengthening the internal validity of our findings.

Conversely, several limitations warrant consideration. The primary limitation stems from the cross-sectional nature of the study design, which precludes the establishment of temporality and causal inference between EASIX and CMM, leaving the possibility of reverse causality. Second, although we adjusted for a comprehensive set of potential confounders, the possibility of residual confounding cannot be entirely ruled out. Third, the definition of CMM partially relied on self-reported history of MI and stroke, which is susceptible to recall bias and misclassification. Fourth, the components of EASIX (LDH, creatinine, platelets) are nonspecific markers that can be influenced by various conditions other than endothelial dysfunction, such as liver disease, muscle injury, or hematological disorders. This lack of specificity might influence the observed association.

Furthermore, while the ROC analysis demonstrated a statistically significant predictive ability for EASIX, it is important to acknowledge its clinical limitations. The optimal cutoff point yielded a modest sensitivity of 43%, implying that it would fail to identify more than half of the true CMM cases. This suggests that EASIX may not be suitable as a standalone screening tool for CMM. Instead, its utility may lie in its combination with other established risk factors to improve overall risk stratification.

## 5. Conclusion

In conclusion, this study demonstrates that the EASIX is significantly and independently associated with an increased risk of CMM in American adults. Future prospective cohort studies and mechanistic investigations are warranted to confirm the causal nature of this association and to elucidate the underlying biological pathways, thereby informing early risk stratification and precise preventive strategies for CMM.

## Acknowledgments

We are grateful to the National Health and Nutrition Examination Survey (NHANES) staff and participants for their valuable contributions, particularly for providing data licenses.

## Author contributions

**Conceptualization:** Qiang Shi.

**Data curation:** Qiang Shi.

**Formal analysis:** Qiang Shi, Xiang Chen, Yifei Chen.

**Funding acquisition:** Xiang Chen, Yifei Chen.

**Investigation:** Xiang Chen, Yifei Chen.

**Methodology:** Qiang Shi, Xiang Chen, Yifei Chen.

**Project administration:** Qiang Shi, Yifei Chen.

**Resources:** Yifei Chen.

**Writing – original draft:** Qiang Shi, Yifei Chen.

**Writing – review & editing:** Qiang Shi, Yifei Chen.

## Supplementary Material


