# Supplementary material for: Association between the endothelial activation and stress index and cardiometabolic multimorbidity: Results from the NHANES 2003 to 2020
Source: Medicine (Baltimore). 2026 Apr 10;105(15):e48226. doi: 10.1097/MD.0000000000048226 (PMC13593069; doi:10.1097/MD.0000000000048226)
Supplement: Supplementary file 1 [file medi-105-e48226-s001.pdf]

Supplement Table 1. Classification and definition of covariates.

Covariate Category	Covariate Name	Categories or source
Sociodemographic Variables	Sex	Male, Female
	Age	From the demographic information obtained in survey questionnaire inquiries
	Race/ethnicity	Mexican American, Other Hispanic, Non-Hispanic White, Non-Hispanic Black, Other Race (including multi-racial)
	Educational Level	Less than high school, High school graduate/GED or equivalent, Higher than high school
	Poverty-to-Income Ratio (PIR)	≤1.3, 1.3–3.5, >3.5
	Marital Status	Married/Living with a partner, Widowed/Divorced/Separated, Never married
Life behavior and disease Variables	Body Mass Index (BMI)	Weight (kg) divided by the square of height (m^2)
	Smoking Status	Nonsmokers (<100 cigarette in lifetime), Former smokers (>100 cigarette in lifetime but no cigarette in current), Current smokers (>100 cigarette in lifetime and cigarette in current)
	Drinking Status	Nondrinkers (no alcohol), Moderate drinkers (≤2 drinks/day for male, ≤1 drink/day for female), Heavy drinkers (>2 drinks/day for male, >1 drink/day for female) in last 12 months
	Hypertension	Defined as meeting any of the following criteria: (1) self-reported history, (2) antihypertensive medication use, or (3) systolic/diastolic blood pressure ≥ 140/90 mm Hg.
	Hyperlipidemia	Defined as meeting any of the following criteria: low density lipoprotein cholesterol (LDL-C) ≥ 130 mg/dL, total cholesterol (TC) ≥ 200 mg/dL, (Triglyceride) TG ≥ 150 mg/dL, or high density lipoprotein cholesterol (HDL-C) ≤ 50 mg/dL in females and ≤ 40 mg/dL in

		males
	Hyperuricemia	Serum uric acid level of ≥ 7 mg/dL in males and ≥ 6 mg/dL in female
